# Comparison of clinicopathological characteristics and prognosis between primary squamous cell carcinoma of the thyroid and squamous cell carcinoma combined with papillary thyroid carcinoma

**DOI:** 10.3389/fendo.2024.1514268

**Published:** 2025-01-15

**Authors:** Wanyun Yan, Huiying Chen, Xiaoyu Lin, Ruifa Zhou, Feng Zhao, Jiping Su

**Affiliations:** Department of Otolaryngology-Head and Neck Surgery, The First Affiliated Hospital of Guangxi Medical University, Nanning, China

**Keywords:** thyroid, primary, squamous cell carcinoma, papillary thyroid carcinoma, clinical features, prognosis

## Abstract

**Background:**

Primary squamous cell carcinoma of the thyroid (PSCCT) has recently been reclassified as a morphologic pattern of anaplastic thyroid carcinoma (ATC). Consequently, PSCCT and squamous cell carcinoma with papillary thyroid carcinoma (SCC-PTC) were categorized as ATC. However, in terms of clinical characteristics and overall prognosis, whether PSCCT is similar to SCC-PTC has yet to be sufficiently investigated. Therefore, this study aimed to elucidate the differences and similarities between PSCCT and SCC-PTC regarding clinicopathological characteristics and prognosis.

**Methods:**

We retrospectively reviewed the medical records of patients with squamous cell carcinoma of the thyroid in our institution from December 2009 to December 2020. In addition, the publications in CNKI, Wanfang, VIP, PubMed, Embase, Web of Science, and ProQuest databases were systematically searched to collect patient information. According to pathological diagnosis, patients were divided into the PSCCT and SCC-PTC groups, and compared their clinical characteristics, treatment, and prognosis, respectively.

**Results:**

308 patients in the PSCCT group and 60 patients in the SCC-PTC group were enrolled in the study. There were significant differences in gender, age, T stage, N stage, M stage, symptoms at diagnosis, and TTF-1 expression between the two groups. Patients in the SCC-PTC group with more frequent radioactive iodine therapy, surgery, and less frequent radiotherapy than PSCCT. In addition, PSCCT and SCC-PTC also demonstrated similarities in tracheal invasion, esophageal invasion, CK5/6 expression, TG expression, P53 expression, and chemotherapy frequency. The 3-year overall survival rate of PSCCT (19.1%) was lower than that of SCC-PTC (34.6%). The prognostic factors were different between the two groups. Multivariable analysis shows that the N stage, M stage, radiotherapy, and tracheal invasion were related to the prognosis of PSCCT, while only the T stage was associated with the prognosis in SCC-PTC.

**Conclusions:**

Clinicopathological characteristics and prognosis were not identical in patients with SCC-PTC and PSCCT. These findings indicated that different clinical treatment and management plans are required for patients with these two types of thyroid cancer.

## 1 Introduction

Primary squamous cell carcinoma of the thyroid (PSCCT) is a highly aggressive malignant tumor, accounting for only 0.12~0.4% of all thyroid cancer (1, 2). Patients with PSCCT often present at an advanced stage due to the rapid tumor progression. The median survival of patients is 7.7 to 9.1 months (1, 3). The classification of PSCCT has evolved over time. Initially categorized as a malignant epithelial tumor in the WHO classification of 1988 (4), it later became recognized as a separate entity of thyroid cancer exclusively characterized by squamous differentiation in the classifications of 2004 and 2017 (4). However, in the recent classification (2022), PSCCT has changed from a separate entity to a morphologic pattern of ATC (5, 6). This change was mainly based on the research of Xu and his colleagues in 2020, which found no significant differences in prognosis and BRAF^V600E^ mutation among PSCCT, ATC having squamous carcinoma component, and other histological types of ATC (7). However, Ye and colleagues in 2024 demonstrated 2047 differential expressed genes between PSCCT and ATC by whole exome sequencing and RNA sequencing, which revealed that PSCCTs exhibited molecular genetic characteristics distinct from ATCs (8). In addition, many studies have highlighted different clinical characteristics and prognoses between ATC and PSCCT. For instance, compared to ATCs, PSCCT tumors are smaller, with less frequent extrathyroidal extension, lymphovascular invasion, and positive margins (1, 4, 8–10). Therefore, there remains controversy regarding whether PSCCT should be classified solely as a morphologic pattern within ATC.

Similarly, PSCCT and SCC-PTC being considered as the same entity remains controversial. A study by Xu and his colleagues in 2020 showed that there was no significant difference in prognosis between PSCCT and squamous cell carcinoma with differentiated thyroid carcinoma (7). However, Lam’s study in 2020 confirmed that patients with PSCCT had a poorer prognosis than patients with well-differentiated thyroid carcinoma (primarily papillary thyroid carcinoma) having a squamous carcinoma component (median survival, 8 months vs 18 months) (4). Due to the rarity of this disease, comparisons of clinical characteristics and prognosis between PSCCT and PTC-SCC are limited. Therefore, this study retrospectively analyzed the clinicopathological characteristics and prognosis of patients with PSCCT, and compared them with patients with SCC-PTC, to comprehensively understand the similarities and differences between them.

## 2 Methods

### 2.1 Inclusion/exclusion criteria

Inclusion criteria: Core-needle biopsy/fine-needle aspiration biopsy or postoperative histopathology diagnosis of squamous cell carcinoma in the thyroid. Exclusion criteria (1): Metastatic or secondary squamous cell carcinoma according to histopathology; (2) Incomplete clinical information regarding gender and age; (3) Whether other types of thyroid cancer coexist is unclear in pathology.

### 2.2 Patient demographics

Institutional review board approval was obtained from The First Affiliated Hospital of Guangxi Medical University on March 28, 2022 (No. 2022-KY-E-086). All patients or their dependents provided written informed consent to participate in the study. With approval from our institutional review board, we conducted a retrospective review of medical records in our institute from December 2009 to December 2020.

### 2.3 Search strategy

A systematic search was conducted of CNKI, VIP, Wanfang, PubMed, Web of Science, Embase, and ProQuest databases (from inception to December 2021) for publications using the keywords “primary,” “cancer,” “squamous,” “neoplasm,” “carcinoma,” and “thyroid”. There were no language restrictions. The complete search strategy is presented in **Supplementary Table 1**.

### 2.4 Tumor case cohort

Patients with pathological diagnosis of pure squamous cell carcinoma were divided into the PSCCT group, while patients with squamous cell carcinoma combined with papillary thyroid carcinoma were classified into the SCC-PTC group.

Patients stage according to the American Joint Committee on Cancer 8 (2018). The lymph node status criteria of surgical patients were grouped according to the pathological results and surgical records. N1 group: lymph nodes with metastasis diagnosed by postoperative pathology. N0 group: lymph node without metastasis was diagnosed by postoperative pathology or the literature records. For non-surgical patients, lymph node grouping was based on imaging results. The M stage criteria are based on clinical staging, while tracheal invasion and esophageal invasion were determined by the surgical records.

The treatment modalities for patients include surgery, radiotherapy, chemotherapy, radioactive iodine therapy, molecular targeted therapy, and immunotherapy. Surgical treatment is the preferred option for the majority of patients, while some literatures only provides a brief overview of surgical interventions without detailing specific treatment histories or subsequent therapies. In the above circumstance, we categorized these patients as belonging to the unknown group.

The overall survival (OS) time is defined as the duration from the initial diagnosis or postoperative to death. According to the literature, we record the time and survival outcome of the patient from the last follow-up.

### 2.5 Immunohistochemistry

During the retrospective review, we collected all the markers for each patient who underwent immunohistochemical testing. Then, we summarized and selectively analyzed some commonly used markers in immunohistochemistry (IHC). The sensitive markers for diagnosing squamous cell carcinoma of the thyroid are P63, P40, Cytokeratin 5/6, and P53. For determining the origin of thyroid follicular epithelium, the markers thyroglobulin (TG), thyroid transcription factor-1 (TTF-1), and Cytokeratin(CK) are frequently utilized. Calcitonin and synaptophysin are employed for distinguishing medullary thyroid carcinoma. Additionally, CD5 is used to differentiate from intrathyroid thymic carcinoma. CK20 is frequently expressed in the gastrointestinal tract to exclude secondary thyroid squamous cell carcinoma. Ki-67 index is a commonly used IHC marker for cell proliferation (4, 11, 12).

### 2.6 Statistical analysis

Categorical variables were expressed as numbers and frequencies as percentages. Descriptive statistics were performed using Pearson’s Chi-squared test, continuity (Yates) correction, and Fisher’s exact test as appropriate. Survival analysis was performed using Kaplan–Meier estimates. A univariable Cox proportional hazards regression analysis on potential factors was performed, and factors identified as statistically significant were subsequently included in a multivariable analysis to identify independent predictors of overall survival in patients with PSCCT or SCC-PTC. Two-sided p values<0.05 were considered statistically significant. All statistical analyses were performed by the Statistical Package for the Social Sciences (SPSS) version 26 (IBM).

## 3 Results

**Figure 1:**
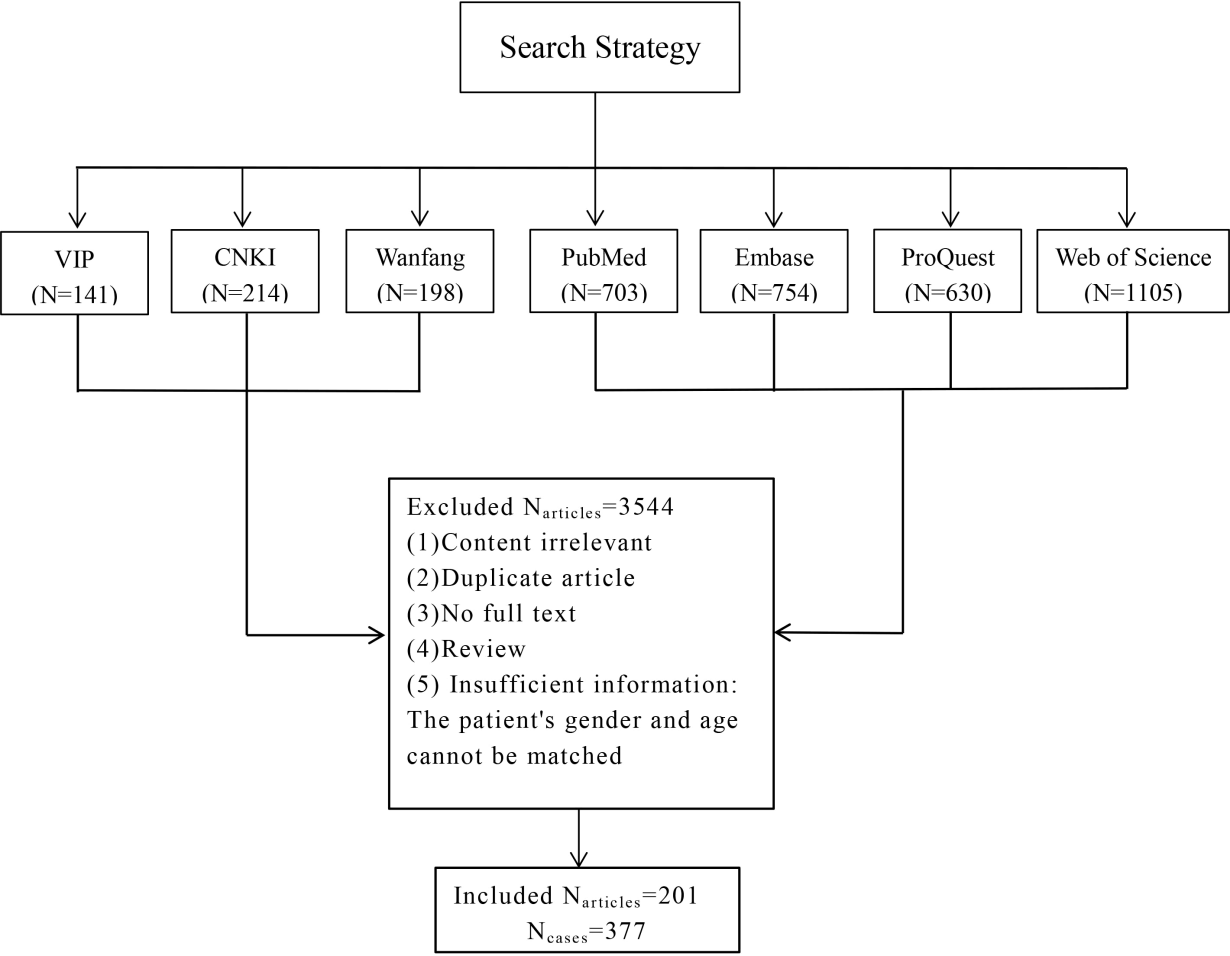
Literature search and study diagram.

There were 11 patients collected in our institute, including 10 patients with pure squamous cell carcinoma and one patient with squamous cell carcinoma coexisting with papillary thyroid carcinoma. 201 papers were selected by the inclusion and exclusion criteria, and these papers contributed information for 377 patients (**Figure 1**). These papers were published from 1971 to 2021. Of the 377 thyroid carcinomas with squamous differentiation collected in the literature in this study, 79% (n = 298) were pure squamous cell carcinomas, while 15.6%(n=59) had coexisting papillary thyroid carcinoma. The remaining 5.4% (n=20) encompassed patients with follicular thyroid carcinoma or other types. Complete article information was presented in **Supplementary Table 2**. Overall, there were 308 patients with PSCCT and 60 with SCC-PTC.

Analysis of differences in demographic and clinical data of patients with SCC-PTC and PSCCT is presented in **Table 1**. In contrast to PSCCT, SCC-PTC has a higher proportion of female patients (78.3% vs. 55.8%). The age of patients with PSCCT ranged from 13 to 91, the median age was 61, and 56% of patients presented at age 60 or above. In SCC-PTC, patients ranged from 24 to 94 years old, with a median age of 68.5 years, and 70% were aged 60 or above. The two groups had a significant difference in age and male-to-female ratio (p<0.05). Additionally, there was a significant difference between the two groups in T1 staging (p<0.05), while there was no difference among T2, T3, T4, and Tx staging. Compared with the PSCCT, SCC-PTC patients had a higher proportion of no distant metastasis (61% vs. 38%, p<0.05) and a lower proportion of unknown distant metastasis (30% vs. 54%, p<0.05). The two groups had similar invasion characteristics to the adjacent structures, manifested in their similar frequency of invading the trachea and esophagus. However, it is worth noting that the proportion of lymph node metastasis in the SCC-PTC was significantly higher than in the PSCCT (63% vs. 28%, p<0.05).

**Table 1:** Clinicopathological characteristics of PSCCT and SCC-PTC.

Characteristic	PSCCT (n = 308) No. (%)	SCC-PTC (n=60) No. (%)	*P value*
Gender			
Male	136 (44.2%)	13 (21.7%)	0.001^1^
Female	172 (55.8%)	47 (78.3%)	
Age (year)			
<40	13 (4.2%)	7 (11.7%)	
40~59	122 (39.6%)	11 (18.3%)	0.001^1^
≥60	173 (56.2%)	42 (70%)	
T stage			
T1	3 (1.0%)	5 (8.3%)	
T2	21 (6.8%)	2 (3.3%)	
T3	59 (19.1%)	7 (11.7%)	0.007^3^
T4	108 (35.1%)	17 (28.4%)	
Tx	117 (38%)	29 (48.3%)	
N stage			
N1	87 (28.2%)	38 (63.3%)	
N0	59 (19.2%)	7 (11.7%)	<0.001^1^
Nx	162 (52.6%)	15 (25%)	
M stage			
M0	117 (38%)	37 (61.7%)	
M1	24 (7.8%)	5 (8.3%)	0.002^1^
Mx	167 (54.2%)	18 (30%)	
Tracheal invasion			
Yes	47 (15.3%)	10 (16.7%)	
No	96 (31.2%)	20 (33.3%)	0.878^1^
Unknown	165 (53.5%)	30 (50%)	
Esophageal invasion			
Yes	26 (8.4%)	5 (8.3%)	
No	117 (38%)	25 (41.7%)	0.862^1^
Unknown	165 (53.6%)	30 (50%)	

^1^Pearson’s Chi-squared test, ^3^Fisher’s exact test.

In terms of clinical presentation, patients with PSCCT or SCC-PTC typically present with a neck mass that invades adjacent structures, leading to obstructive symptoms caused by the mass effect of the tumor, such as dysphagia, dyspnea, and hoarseness. Compared to the SCC-PTC cohorts, patients with PSCCT had a higher incidence of dysphagia (29% vs. 10%, p<0.05) and hoarseness (46% vs. 15%, p<0.05). However, the two groups had no significant difference in non-specific symptoms, including cough, pharyngalgia, and neck pain (**Table 2**).

**Table 2:** Analysis of differences in symptoms between PSCCT and SCC-PTC.

Symptoms	PSCCT (n = 228) No. (%)	SCC-PTC (n = 40) No. (%)	*P value*
Neck mass	171 (75%)	33 (82.5%)	0.305^1^
Hoarseness	107 (46.9%)	6 (15%)	<0.001^1^
Dysphagia	67 (29.4%)	4 (10%)	0.010^1^
Neck pain	45 (19.7%)	3 (7.5%)	0.063^1^
Dyspnoea	67 (29.4%)	6 (15%)	0.059^1^
Cough	19 (8.3%)	2 (5%)	0.686^2^
Pharyngula	7 (3.1%)	1 (2.5%)	1.000^2^

^1^Pearson’s Chi-squared test, ^2^Continuity (Yates) correction.

A significantly higher proportion of patients with SCC-PTC underwent radioactive iodine therapy (27% vs. 0%, p<0.05) and surgery (100% vs. 84%, p<0.05), whereas radiation therapy was more prevalent in the PSCCT group (63% vs. 46%, p<0.05). However, there was no significant difference in the utilization of chemotherapy and molecular targeted (immune) therapy between the two groups (**Table 3**).

**Table 3:** Frequency of treatments chosen in patients with PSCCT and SCC-PTC.

Therapeutic Modalities	PSCCT (n = 206) No. (%)	SCC-PTC (n = 43) No. (%)	*P value*
Surgery	174 (84.5%)	43 (100%)	0.006^1^
Radiotherapy	130 (63.1%)	20 (46.5%)	0.043^1^
Chemotherapy	73 (35.4%)	12 (27.9%)	0.344^1^
Radioactive iodine therapy	0 (0%)	12 (27.9%)	<0.001^2^
Molecular targeted (immune) therapy	8 (3.9%)	5 (11.6%)	0.089^2^

^1^Pearson’s Chi-squared test, ^2^Continuity (Yates) correction.

There were no differences in the expression of CK5/6, TG, and P53 between the two groups, whereas the expression of TTF-1 in the SCC-PTC was significantly higher than the PSCCT (55.6% vs. 6.5%, p<0.05) (**Table 4**). The PSCCT group included 24 patients with recorded Ki-67 index, ranging from 20% to 90% and an average Ki-67 index of 50%. Furthermore, the positive expression rates of CK (20/21), P63 (47/48), and P40 (19/20) were all 95% or above in PSCCT, while CK20 (27/27), calcitonin (41/41), synaptophysin (13/13), and CD5 (33/33) were negative. Similarly, the expression for CK (3/3), P63 (9/9), and P40 (3/3) in the SCC-PTC group demonstrated positive, while the expression of CD5 (4/4) and calcitonin (4/4) was negative. Further analysis was not conducted due to the limited data available for synaptophysin and CK20 in the SCC-PTC group.

**Table 4:** Pathological characteristics between the PSCCT and SCC-PTC group.

Immunohistochemistry	Positive (No, %)	Negative (No, %)	Total No.	*P value*
TG PSCCT SCC-PTC	2 (2.7%) 2 (14.3%)	71 (97.3%) 12 (85.7%)	73 14	0.120^3^
TTF-1 PSCCT SCC-PTC	4 (6.5%) 5 (55.6%)	58 (93.5%) 4 (44.4%)	62 9	<0.001^2^
P53 PSCCT SCC-PTC	22 (84.6%) 8 (80%)	4 (15.4%) 2 (20%)	26 10	1.000^3^
CK5/6 PSCCT SCC-PTC	43 (97.7%) 11 (100%)	1 (2.3%) 0 (0%)	44 11	1.000^3^

^2^Continuity (Yates) correction, ^3^Fisher’s exact test.

**Figure 2:**
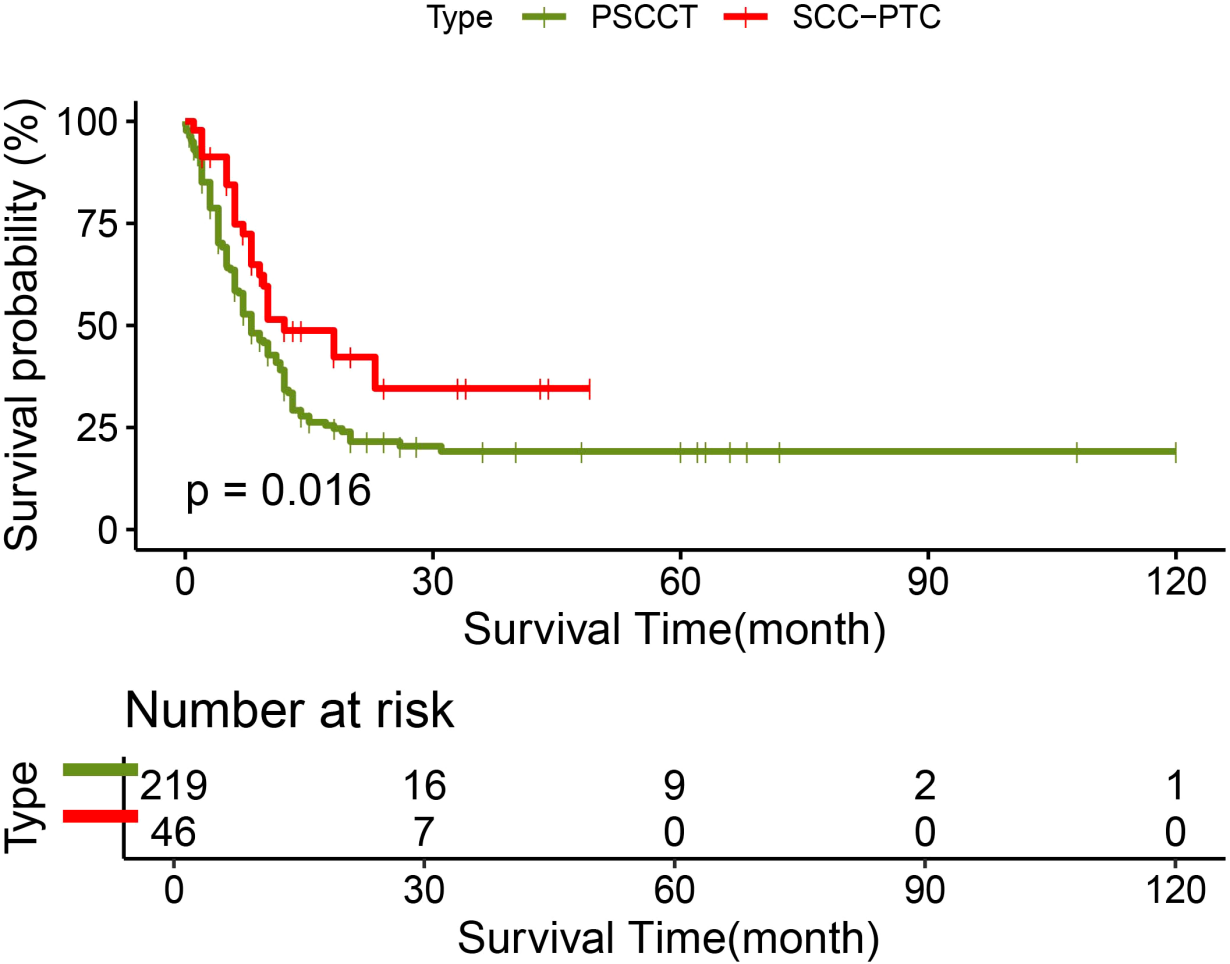
Survival curve in PSCCT group and SCC-PTC group.

219 patients in the PSCCT and 46 patients in the SCC-PTC group had recorded follow-up times. In the PSCCT cohort, the 1-, 2-, and 3-year OS were 34.2%, 21.5%, and 19.1% respectively, with a median survival time of 8 months. On the other hand, the 1-, 2-, and 3-year OS of SCC-PTC were 48.8%, 34.6%, and 34.6% respectively, with a median survival time of 12 months. The log-rank test demonstrated that the OS of SCC-PTC patients was better than PSCCT patients (P<0.05) (**Figure 2**).

**Figure 3:**
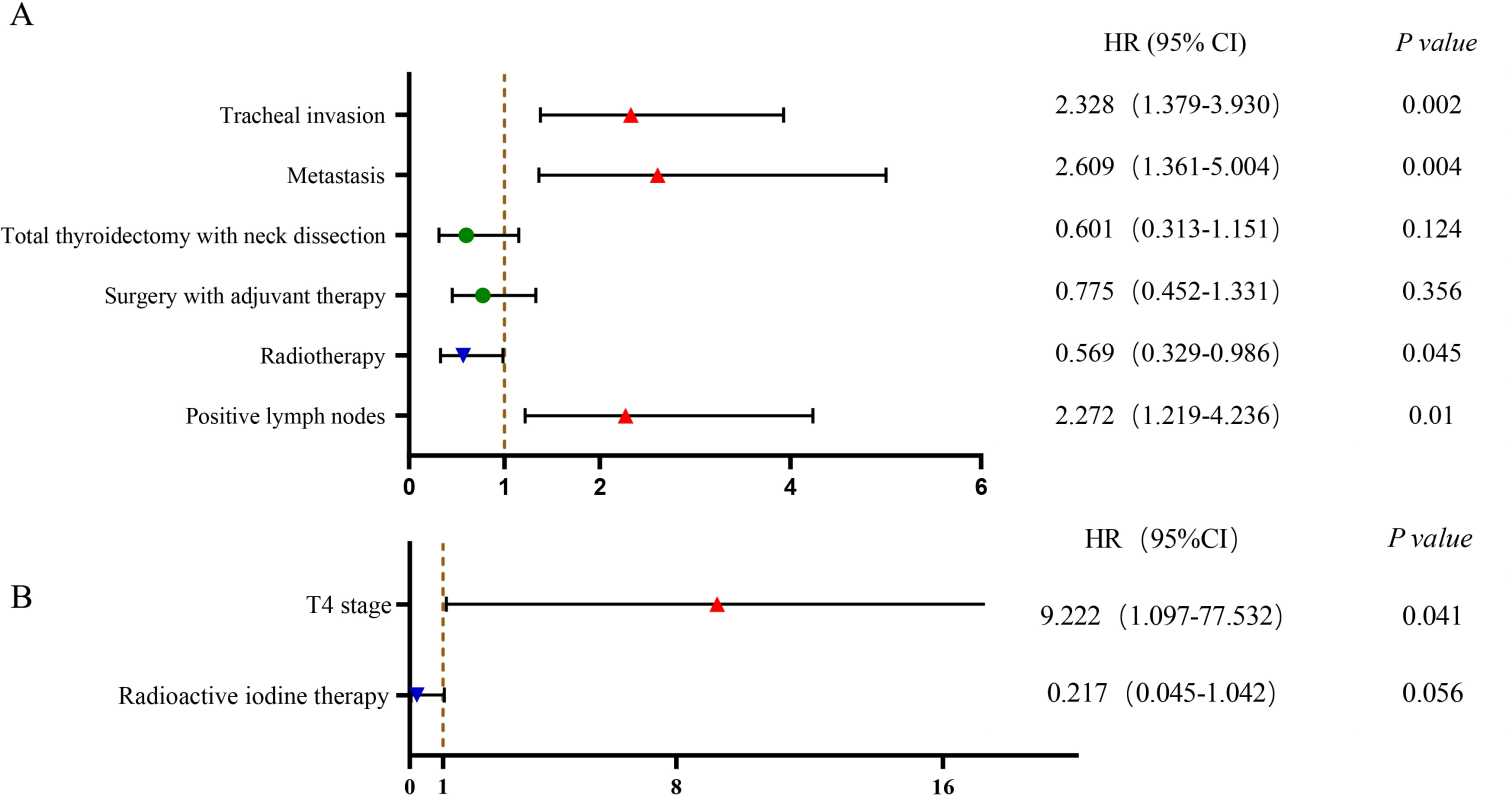
Multivariable Cox regression of factors associated with OS in PSCCT and SCC-PTC: **(A)** PSCCT group; **(B)** SCC-PTC group.

An analysis of PSCCT patients was performed to identify independent factors impacting OS. We conducted univariate and multivariate Cox regression on potential prognostic factors. Univariate analysis showed that N stage, M stage, tracheal invasion, radiotherapy, surgery combined with adjuvant therapy, and total thyroidectomy with neck lymph node dissection were associated with OS (P<0.05). Age, gender, chemotherapy, esophageal invasion, and T4 stage did not show statistically significant differences in predicting OS in PSCCT. Multivariate analysis demonstrated that radiotherapy improved prognosis [HR 0.569 (95% CI 0.329-0.986), p<0.05]. Conversely, distant metastasis [HR 2.609 (95% CI 1.361-5.004), p<0.05], tracheal invasion [HR 2.328 (95% CI 1.379-3.930), p<0.05], and lymph node metastases [HR 2.272 (95% CI 1.219-4.236), p<0.05] were associated with worse OS in PSCCT patients. To compare the factors influencing the prognosis of SCC-PTC patients, we also performed univariate and multivariate Cox model analyses on potential factors. Univariate Cox regression revealed that T staging and radioactive iodine therapy were significant factors affecting the OS of SCC-PTC. Multivariate analysis showed that patients with T4 staging had a poor prognosis [HR 9.222 (95% CI 1.097-77.532), p<0.05] (**Table 5**; **Figure 3**).

**Table 5:** Univariate Cox regression of factors associated with OS in patients with PSCCT and SCC-PTC.

Characteristic	PSCCT (N=219)		SCC-PTC (N=46)	
	HR (95%CI)	*P value*	HR (95%CI)	*P value*
Gender				
Male Female	Reference 1.074 (0.773-1.492)	0.671	Reference 0.965 (0.384-2.426)	0.940
Age(year) <65 ≥65	Reference 1.385 (0.992-1.993)	0.056	Reference 1.386 (0.612-3.138)	0.434
N stage N0 N1 Nx	Reference 2.803 (1.527-5.145) 2.744 (1.557-4.837)	0.001 <0.001	Reference 2.252 (0.519-9.761) 3.469 (0.697-17.265)	0.278 0.129
Surgery with adjuvant therapy No Yes Unknown	Reference 0.538 (0.374-0.774) 1.078 (0.666-1.743)	0.001 0.760	Reference 0.963 (0.320-2.894) 3.109 (0.820-11.792)	0.946 0.095
Total thyroidectomy with neck dissection No Yes Unknown	Reference 0.384 (0.211-0.700) 0.962 (0.637-1.454)	0.002 0.856	Reference 0.509 (0.207-1.251) 1.683 (0.543-5.220)	0.141 0.367
Chemotherapy No Yes Unknown	Reference 0.952 (0.653-1.387) 1.635 (1.043-2.561)	0.796 0.032	Reference 1.250 (0.499-3.137) 3.450 (1.213-9.807)	0.634 0.020
Radiotherapy No Yes Unknown	Reference 0.492 (0.339-0.713) 1.026 (0.633-1.663)	<0.001 0.917	Reference 1.473 (0.600-3.617) 3.784 (1.285-11.148)	0.398 0.016
Esophageal invasion No Yes Unknown	Reference 1.524 (0.853-2.723) 1.523 (1.065-2.177)	0.154 0.021	Reference 2.627 (0.792-8.715) 1.124 (0.473-2.669)	0.114 0.792
Tracheal invasion No Yes Unknown	Reference 1.883 (1.135-3.125) 1.715 (1.167-2.521)	0.014 0.006	Reference 2.272 (0.761-6.787) 1.231 (0.484-3.131)	0.142 0.662
M stage M0 M1 Mx	Reference 2.999 (1.616-5.565) 1.943 (1.364-2.769)	<0.001 <0.001	Reference 1.633 (0.468-5.694) 4.664 (1.887-11.525)	0.442 0.001
Radioactive iodine therapy No Yes Unknown	N/A N/A N/A	N/A N/A N/A	Reference 0.201 (0.047-0.871) 2.333(0.856-6.359)	0.032 0.098
T stage T1-T3 T4 Tx	Reference 1.215 (0.775-1.906) 1.571 (1.023-2.412)	0.396 0.039	Reference 15.357 (1.958-120.471) 6.773 (0.886-51.810)	0.009 0.065

N/A, not available.

## 4 Discussion

To our knowledge, our study is the first to illustrate SCC-PTC patients’ unique clinical and pathological characteristics that are discrepant from PSCCT. PSCCT and SCC-PTC patients’ clinical characteristics and prognoses may be different according to their tumor components. Therefore, it is necessary to conduct more comparative studies between both. Our study demonstrated that the 3-year OS rate of SCC-PTC (34.6%) was better than PSCCT (19.1%, p<0.05), suggesting the specific tumor characteristics of SCC-PTC seem to predict a better prognosis, such as less frequent symptoms of dysphagia and hoarseness at presentation, a higher proportion of M0 stage, and 100% patients received surgical treatment. Regional lymph node positivity was more common in SCC-PTC than PSCCT(63% vs. 28%, p<0.05). Similarly, Yasuhiro studied 10 cases of SCC-PTC and found lymph node metastasis with a papillary carcinoma component in all cases, and two of them contained a squamous cell carcinoma component (13).

Regional lymph node positivity correlated with poorer median survival time in the PSCCT group (31 months vs. 8 months, p<0.05) but not in the SCC-PTC group. To further analyze the underlying reasons for this discrepancy, we reviewed relevant studies and found controversy over whether regional lymph node positivity is related to the prognosis of patients with papillary thyroid carcinoma (PTC). In 2008, Zaydfudim studied 30504 PTC patients and observed that regional lymph node metastasis did not correlate with survival rates in patients younger than 45 years old, but was associated with prognosis in patients aged 45 or above (14). Conversely, a retrospective analysis by Adam in 2015 involving 47902 PTC patients under the age of 45 years indicated that cervical lymph node metastasis was linked to inferior survival outcomes (15). In addition, studies examining the association between the number of involved lymph nodes and disease outcomes have been inconsistent. One review identified structural disease recurrence rates of more than 20% to 30% were seen in large-volume lymph node metastases (>3 cm or >5–10 involved lymph nodes) (16). Another study of patients aged <45 years showed that the number of involved nodes ≤6 nodes was associated with reduced survival, but no additional mortality risk was observed with >6 nodes (15). Therefore, in our PTC-SCC group, we cannot definitively state that positive regional lymph nodes impact prognosis. We plan to conduct stratification and subgroup analysis to address this issue in the future.

Uniquely, thyroid cancer is the only non-reproductive cancer with striking female predominance, with a 3-4-fold higher incidence among females (17, 18). Previous studies showed that the male-to-female ratio of PSCCT ranges from 1:1.37 to 1:2.4 (2, 3). In our study, the ratio of male-to-female was 1:1.26 in the PSCCT. The ratio of male-to-female in the SCC-PTC group was 1:3.61, which more closely resembled differentiated thyroid carcinoma. Some retrospective studies have concluded that gender does not impact the prognosis of differentiated thyroid carcinoma despite the female predominance of thyroid cancer (18). Similarly, we observed that gender did not influence the prognosis of either the PSCCT or SCC-PTC groups.

Immunohistochemistry helps identify whether a tumor is primary or secondary to the thyroid, diagnose uncommon thyroid cancer subtypes, evaluate BRAF^V600E^ mutations, provide prognostic information, and predict response to treatment (19). The common immunohistochemistry markers for diagnosing PSCCT or SCC-PTC include TG, TTF-1, CK5/6, P53, P40, P63, CK, and calcitonin. CK5/6 is overexpressed in squamous cell carcinoma and cannot be detected in normal thyroid parenchyma, lymphocytic thyroiditis, follicular carcinoma, and poorly differentiated carcinoma (20, 21). We found that CK5/6 was expressed in over 97% of PSCCT patients (43/44), which is consistent with the results reported by Lam AK(14/14,100%) (4). Calcitonin is a 32-amino acid monomeric peptide secreted by parafollicular C cells and highly expressed in medullary thyroid carcinoma (22). Our study showed that all patients with PSCCT were negative for calcitonin, which is similar to Booya F’s study (23). Calcitonin may aid in distinguishing medullary thyroid carcinoma from PSCCT.

TTF-1 mediates cell determination and differentiation in the thyroid and expresses in normal thyroid follicular and parafollicular cells. Loss of TTF-1 causes differentiated or poorly differentiated thyroid cancer to lose the ability to absorb, concentrate radioactive iodine, and produce thyroglobulin during progression (24–26).In previous studies, it expressed nearly 100% of PTC and follicular thyroid carcinomas (27). Only approximately 5.7% to 18% of ATCs are TTF-1 positive, and the rate in PSCCT is about 17% (4, 28, 29). We demonstrated that 6.5% (4/62) of PSCCT patients express TTF-1, while 55.6% (5/9) of SCC-PTC patients were positive. There was a significant difference in TTF-1 expression between both. According to its pathological features, the loss of TTF-1 may be a characteristic of PSCCT: the normal follicular thyroid cells had undergone squamous metaplasia. TG is secreted by thyroid follicular cells and is a precursor protein for thyroid hormone synthesis (30). In both PTC and follicular thyroid carcinoma, TTF-1 expression has been reported in 95% or above, whereas approximately 7.5% is expressed in ATC (30). We reported TG expression in 2.7% (2/73) of PSCCT patients, whereas 14.3% is represented in SCC-PTC. We hypothesize that PSCCT cannot maintain normal follicular cells’ biological characteristics and functions, leading to low TG expression. Conversely, the increasing levels of TG and TTF-1 observed in the SCC-PTC group may be associated with histologic coexisting PTC.

At present, there is still no unified treatment approach for PSCCT patients. It is challenging in clinical settings to quickly and accurately select individualized treatment for PSCCT patients, depending on their condition. The guidelines from the National Comprehensive Cancer Network (NCCN) in 2024 recommend that ATC patients consider total thyroidectomy with therapeutic neck lymph node dissection following: 1. Patients with stage IVA/IVB ATC achieve negative(R0) or microscopic (R1) surgical tumor margins; 2. IVC patients who expected aggressive therapy and R0/R1 resection are feasible (31). Limberg (1) showed that successful surgical resection of PSCCT tumor could improve median OS (complete microscopic resection: 55.5 months vs. complete macroscopic resection: 10.2 months vs. incomplete macroscopic resection: 3.4 months, p<0.001). However, in the multivariate analysis of PSCCT, we could not observe that total thyroidectomy with neck lymph node dissection was associated with prognosis. We analyzed that it may be attributed to certain surgeries not achieving R0/R1 resection even though the preoperative goal was to perform total thyroidectomy with therapeutic neck lymph node dissection. Because the data collected by our study cover decades and a broad regional distribution and different levels of surgical proficiency. Given the characteristic rapid progression of PSCCT, certain patients typically present with a thyroid mass that invades the larynx, strap muscles, trachea, esophagus, and major neck vessels (32, 33). In clinical practice, the extent of surgical resection often involves not only a total thyroidectomy combined with neck lymph node dissection but also an extension to include the larynx, trachea, and esophagus. In some cases, free flap reconstruction may be considered for reconstruction (32). When performing these extended operations, it is crucial to assess the potential for complications such as cervical hematoma, wound infection, and laryngeal nerve paralysis (34, 35). These serious complications might delay the patient’s subsequent radiotherapy and systemic chemotherapy (36).

Radiation therapy is still a mainstream treatment of ATC (37). The NCCN guidelines in 2024 recommend ATC patients consider radiotherapy the following (31): 1. ATC patients obtaining an R2 resection(grossly positive margins or debulking operations) in stage IVA/IVB; 2. ATC patients unresected in stage IVA/IVB; 3. Adjuvant radiotherapy in ATC patients with R0/R1 resection; 4. Local radiotherapy for ATC patients in stage IVC. Nevertheless, there are inconsistent studies on the prognosis and adjuvant radiotherapy of PSCCT. Au JK (3) demonstrated radiotherapy cannot improve the prognosis of PSCCT patients. In Limberg’s study, neither adjuvant radiation nor chemotherapy showed any survival benefit when at least a complete macroscopic resection was achieved. In contrast, ATC patients with complete macroscopic resection had prolonged 5 months in median survival when treated with adjuvant radiotherapy, chemotherapy, or chemoradiotherapy (1). Our study observed radiotherapy prolongs the median survival in PSCCT(11.5 months vs.4 months).

Many studies have demonstrated that chemotherapy is not associated with prognosis in PSCCT (38, 39). It is not recommended treatment for PSCCT due to adverse reactions. Our study revealed that chemotherapy did not improve the prognosis in PSCCT, which is consistent with previous reports. Along with the development of molecular targeted therapy, it is also described in PSCCT. Mary Torrez (40) first reported a PSCCT patient with BRAF^V600E^ mutated and started on dabrafenib plus trametinib treatment, but the outcome was unsatisfactory. However, Brandenburg T demonstrated that a PSCCT patient with BRAF^V600E^ mutated and treated with dabrafenib and trametinib, observing the tumor size decreased and had a good quality of life within one year (41). In conclusion, molecular targeted therapy has recently emerged as a novel cancer treatment modality and has exhibited specific efficacy in managing PSCCT. Nevertheless, due to the limited data, further research is warranted to validate its potential benefits.

Besides, we observed significant differences in the treatment involving radioactive iodine therapy between the two groups. Patients with PSCCT did not opt for radioactive iodine therapy, whereas over 25% of patients with SCC-PTC underwent radioactive iodine therapy post-surgery. Additionally, in the multivariate analysis of the SCC-PTC group, there was a trend suggesting that radioactive iodine therapy may improve prognosis [HR 0.217 (95% CI 0.045-1.042) p=0.056]. However, further analysis is necessary.

Due to the rarity of PSCCT, data on patients is limited both in our institute and in the existing literature. Consequently, drawing comprehensive conclusions for a larger population becomes challenging. To address this, our team have tried to collect data from the SEER database. Ten different morphological subtypes of squamous cell carcinoma were identified and coded from 8070/2 to 8078/3 based on ICD-O-3 histology. Unfortunately, no specific coding definition for squamous cell carcinoma with papillary thyroid carcinoma is available. Therefore, future data mining endeavors would be valuable in expanding our understanding. Despite these limitations, our study represents the largest investigation of SCC-PTC regarding its clinical features, treatment, and prognostic factors. It sheds light on the clinicopathological relationship between PSCCT and SCC-PTC.

## 5 Conclusion

PSCCT and SCC-PTC are common in the elderly population, with neck mass being the primary clinical manifestation. For PSCCT patients, lymph node metastasis, distant metastasis, and tracheal invasion were associated with worse OS, whereas radiotherapy improves prognosis. SCC-PTC patients demonstrated a better prognosis than PSCCT, with T4 staging related to poorer outcomes. Furthermore, radioactive iodine therapy appeared to have a tendency to improve the prognosis for SCC-PTC patients.

## Data Availability

The raw data supporting the conclusions of this article will be made available by the authors, without undue reservation.
