# Supplementary material for: Comparison of clinicopathological characteristics and prognosis between primary squamous cell carcinoma of the thyroid and squamous cell carcinoma combined with papillary thyroid carcinoma
Source: Front Endocrinol (Lausanne). 2025 Jan 15;15:1514268. doi: 10.3389/fendo.2024.1514268 (PMC11774738; doi:10.3389/fendo.2024.1514268)
Supplement: Supplementary file 1 [file Table1.docx]

Supplementary Material

## Supplementary Table 1: Search strategy

## Table 1.1 Search strategy in PubMed, Embase, Web of Science, and ProQuest database

| Search | Query( before Dec 22, 2021) | Items found |
| --- | --- | --- |
| PubMed | Search: ((((Thyroid Carcinomas) OR (Thyroid Neoplasm)) OR (Thyroid Cancers)) AND (Squamous Cell Carcinoma)) AND (Primary)  ("thyroid neoplasms"[MeSH Terms] OR ("thyroid"[All Fields] AND "neoplasms"[All Fields]) OR "thyroid neoplasms"[All Fields] OR ("thyroid"[All Fields] AND "carcinomas"[All Fields]) OR "thyroid carcinomas"[All Fields] OR ("thyroid neoplasms"[MeSH Terms] OR ("thyroid"[All Fields] AND "neoplasms"[All Fields]) OR "thyroid neoplasms"[All Fields] OR ("thyroid"[All Fields] AND "neoplasm"[All Fields]) OR "thyroid neoplasm"[All Fields]) OR ("thyroid neoplasms"[MeSH Terms] OR ("thyroid"[All Fields] AND "neoplasms"[All Fields]) OR "thyroid neoplasms"[All Fields] OR ("thyroid"[All Fields] AND "cancers"[All Fields]) OR "thyroid cancers"[All Fields])) AND ("carcinoma, squamous cell"[MeSH Terms] OR ("carcinoma"[All Fields] AND "squamous"[All Fields] AND "cell"[All Fields]) OR "squamous cell carcinoma"[All Fields] OR ("squamous"[All Fields] AND "cell"[All Fields] AND "carcinoma"[All Fields])) AND ("primaries"[All Fields] OR "primary"[All Fields]) | 703 |
| Web of Science | ((((Thyroid Carcinomas) OR (Thyroid Neoplasm)) OR (Thyroid Cancers)) AND (Squamous Cell Carcinoma)) AND (Primary)(Topic) | 1105 |
| ProQuest | su(((((Thyroid Carcinomas) OR (Thyroid Neoplasm)) OR (Thyroid Cancers)) AND (Squamous Cell Carcinoma)) ) OR ab(((((Thyroid Carcinomas) OR (Thyroid Neoplasm)) OR (Thyroid Cancers)) AND (Squamous Cell Carcinoma))) | 630 |
| Embase | ('Thyroid Carcinomas' OR 'Thyroid Neoplasm' /exp OR 'Thyroid Cancers') AND 'Squamous Cell Carcinoma'/exp AND Primary | 754 |

## Table 1.2 Search strategy in CNKI, VIP, and Wanfang database

| Search | Query（ before Dec 22, 2021） | Items  found |
| --- | --- | --- |
| CNKI | 关键词：(甲状腺 * (鳞状细胞癌 + 鳞癌) * 原发性)（精确）） OR 摘要：(甲状腺 * (鳞状细胞癌 + 鳞癌) * 原发性)（精确）） OR 篇名：(甲状腺 * (鳞状细胞癌 + 鳞癌) * 原发性)（精确））  Translation: Keywords: (thyroid* (squamous cell carcinoma + squamous carcinoma) * primary) (exact)) OR abstract: (thyroid * (squamous cell carcinoma + squamous carcinoma) * primary) (exact)) OR Tittle: (thyroid* (squamous cell carcinoma + squamous carcinoma) * primary) (exact)) | 214 |
| VIP | 关键词：((甲状腺 and (鳞状细胞癌 or 鳞癌) and 原发性)） or 摘要： ((甲状腺 and (鳞状细胞癌 or 鳞癌) and 原发性)） or 篇名： ((甲状腺 and (鳞状细胞癌 or 鳞癌) and 原发性)）  Translation: Keywords: (thyroid AND (squamous cell carcinoma OR squamous carcinoma) AND primary)) OR abstract: (thyroid AND (squamous cell carcinoma OR squamous carcinoma) AND primary)) OR Tittle: (thyroid AND (squamous cell carcinoma OR squamous carcinoma) AND primary)) | 141 |
| Wanfang | 关键词：((甲状腺 and (鳞状细胞癌 or 鳞癌) and 原发性)） or 摘要： ((甲状腺 and (鳞状细胞癌 or 鳞癌) and 原发性)） or 篇名： ((甲状腺 and (鳞状细胞癌 or 鳞癌) and 原发性)）  Translation: Keywords: (thyroid AND (squamous cell carcinoma OR squamous carcinoma) AND primary)) OR abstract: (thyroid AND (squamous cell carcinoma OR squamous carcinoma) AND primary)) OR Tittle: (thyroid AND (squamous cell carcinoma OR squamous carcinoma) AND primary)) | 198 |

## Supplementary Table 2. The articles of literature review

Table 2.1 The articles of literature review in CNKI, VIP, and Wanfang database

|  | Year | No. | Journal | Volume (issue) | Page | Author |
| --- | --- | --- | --- | --- | --- | --- |
| 1 | 1981 | 1 | Henan Medical Journal | (6) | 43-44 | Yin PZ |
| 2 | 1982 | 2 | Chinese Journal of Oncology | 4(2) | 142 | Wei JD |
| 3 |  |  | Journal of Medical Science Yanbian University | 5(4) | 48 | Pu DM |
| 4 | 1985 | 1 | Hebei Medical Journal | 7(1) | 40-42 | Wei SL |
| 5 | 1986 | 2 | Shanghai Medical Journal | 9(10) | 613-614 | Gu JS |
| 6 |  |  | Shanghai Medical Journal | 9(9) | 544 | Zhan YH |
| 7 | 1987 | 2 | Chinese Journal of Radiation Oncology | 1(3) | 53 | Gong YR |
| 8 |  |  | Chinese Journal of Cancer | (3) | 191 | Ma NX |
| 9 | 1989 | 5 | Zhejiang Cancer Communication | (4) | 50 | Ni XH |
| 10 |  |  | Zhejiang Medical Journal | 11(4) | 242 | Shi QL |
| 11 |  |  | Chinese Journal of Clinical Oncology | 16(4) | 210-211 | Wu CC |
| 12 |  |  | Practical Oncology Journal | (1) | 70 | Chen KM |
| 13 |  |  | Chinese Journal of Surgery | 27(10) | 584 | Zhang ZC |
| 14 | 1990 | 1 | Chinese Journal of Clinical Oncology | 17(1) | 59-60 | Yang FD |
| 15 | 1991 | 2 | Journal of Dalian Medical University | 13(2) | 69 | Ren LQ |
| 16 |  |  | Qinghai Medical Journal | (5) | 49-50 | Sonam RQ |
| 17 | 1992 | 3 | Journal of Ningxia Medical University | 14(1) | 88 | Ma T |
| 18 |  |  | Tumor | (2) | 95 | Zhang ZJ |
| 19 |  |  | Chinese Journal of Pathology | 21(3) | 155 | Zhang Y |
| 20 | 1993 | 5 | Practical Journal of Medicine & Pharmacy | 6(2) | 52 | Wang SH |
| 21 |  |  | Journal of Practical Oncology | 8(2 ) | 86 | Yan AG |
| 22 |  |  | Henan journal of oncology | (2) | 89 | Xu SH |
| 23 |  |  | Journal of Bengbu Medical College | 18(1) | 42 | Huang MM |
| 24 |  |  | Tumor | (2) | 56 | Liu ZF |
| 25 | 1995 | 1 | Chinese Journal of Diagnostic Pathology | 2(3) | 182 | Pang YC |
| 26 | 1996 | 3 | Henan journal of oncology | 9(2) | 136-137 | Wang XZ |
| 27 |  |  | Cancer Research and Clinic | 8(1) | 31-32 | Zhan YH |
| 28 |  |  | Journal of the First Military Medical University | 16(2) | 150-151 | Zheng ZM |
| 29 | 1998 | 2 | Chinese Journal of Oncology | 20(1 ) | 56 | Wang N |

Table 2.1 The articles of literature review in CNKI, VIP, and Wanfang database (continued 1)

|  | Year | No. | Journal | Volume (issue) | Page | Author |
| --- | --- | --- | --- | --- | --- | --- |
| 30 |  |  | Tumor | 19(4) | 242 | Zhang JW |
| 31 | 1999 | 2 | Chinese Journal of Clinical Oncology | 26(8) | 585 | Zhou B |
| 32 |  |  | Cancer Research and Clinic | 11(5) | 358 | Han DX |
| 33 | 2000 | 4 | Tumor | 20(6) | 463 | Ma JD |
| 34 |  |  | Jilin Medical Journal | (7) | 17 | Li XM |
| 35 |  |  | Chinese Journal of Diagnostic Pathology | 7(2) | 144 | Qin J |
| 36 |  |  | Journal of Medical Graduate Students | 13 | 277-278 | Zhang LH |
| 37 | 2001 | 2 | Chinese Journal of Diagnostic Pathology | 8(1) | 54 | Li KF |
| 38 |  |  | Aviation Medicine of Air Force | 17( 2) | 100 | Zhou XW |
| 39 | 2002 | 4 | West China Medical Journal | 17(4) | 565 | Li J |
| 40 |  |  | Shaanxi Oncology Medicine | 10(3) | 203-204 | Xiao H |
| 41 |  |  | Chinese Journal of Misdiagnostics | 2(7) | 1108 | Liu XB |
| 42 |  |  | China Journal of General Surgery | 17(8) | 483 | Li XM |
| 43 | 2003 | 1 | Practical Journal of Medicine & Pharmacy | 20 (6) | 480 | Dai XQ |
| 44 | 2004 | 1 | Chinese Journal of Clinical Oncology | 31(7) | 364 | Shen XB |
| 45 | 2005 | 1 | Chinese Journal of Current Advances in General Surgery | 8(1) | 62 | Sun QL |
| 46 | 2006 | 5 | Chinese Journal of Clinical Oncology and Rehabilitation | 13(3) | 255 | Li LX |
| 47 |  |  | Cancer Research and Clinic | 18(9) | 624-625 | Shao MH |
| 48 |  |  | Acta Academiae Medicinae Weifang | 28(6) | 471 | Zhang YX |
| 49 |  |  | Journal of Clinical Otorhinolaryngology Head and Neck Surgery | 20(13) | 599 | Li L |
| 50 |  |  | Chinese Journal of Otorhinolaryngology-Skull Base Surgery | 12(2) | 116-117 | Ma J |
| 51 | 2007 | 6 | Journal of Clinical Radiology | 27(7) | 992 | Liang JF |
| 52 |  |  | Journal of Southeast University  (Medical Science Edition) | 26(3) | 213-216 | Xu XY |
| 53 |  |  | Chinese Journal of Clinical and Experimental Pathology | 23(5) | 635 | Zhang GL |
| 54 |  |  | Chinese Archives of Otolaryngology-Head and Neck Surgery | 14(12) | 691-692 | Liu F |
| 55 |  |  | China Journal of General Surgery | (3) | 257 | Zhao DH |

Table 2.1 The articles of literature review in CNKI, VIP, and Wanfang database (continued 2)

|  | Year | No. | Journal | Volume (issue) | Page | Author |
| --- | --- | --- | --- | --- | --- | --- |
| 56 |  |  | China Journal of General Surgery | 16(11) | 1126 | Zhao XY |
| 57 | 2008 | 1 | Chinese Journal of Bases and Clinics in General Surgery | 15(2) | 105 | Ou Y |
| 58 | 2010 | 4 | China Journal of General Surgery | 19(11) | 1259-1260 | Cai XY |
| 59 |  |  | Chinese Journal of Bases and Clinics in General Surgery | 17(8) | 780-782 | Wang J |
| 60 |  |  | Journal of Huaihai Medicine | 28( 3) | 219-220 | Hu JY |
| 61 |  |  | Chinese Journal of Cancer Prevention and Treatment | 17(23) | 1960-1962 | Hu ZX |
| 62 | 2011 | 3 | Chinese Journal of General Practice | 9(4) | 664 | Jiang T |
| 63 |  |  | Chinese Journal of Laboratory Diagnosis | 15(5) | 936-937 | Li SJ |
| 64 |  |  | Hainan Medical Journal | 22(11) | 139 | Xiang BT |
| 65 | 2012 | 2 | Maternal and Child Health Care of China | 36 | 6071 | Yang T |
| 66 |  |  | The 8th National Conference on Integrated Traditional Chinese and Western Medicine Disaster Emergency and Critical Care Medicine | N/A | 296-300 | Wang Y |
| 67 | 2013 | 4 | Chinese Journal of Endocrine Surgery | 7(1) | 80 | Wu ZY |
| 68 |  |  | Chinese Journal of Ultrasonography | 22(10) | 869-872 | Wei L |
| 69 |  |  | Journal of Gannan Medical University | 33(2) | 282 | Xiao H |
| 70 |  |  | Chinese Journal of Clinical Rational Drug Use | 6(3) | 130-131 | Meng G |
| 71 | 2014 | 7 | Progress in Modern Biomedicine | 14(30) | 5884-5887 | Liu Y |
| 72 |  |  | Chinese Journal of the Frontiers of Medical Science | (4) | 253 | He YQ |
| 73 |  |  | Chinese Journal of Practical Surgery | 34(6) | 587-588 | Wu YL |
| 74 |  |  | Journal of Qiqihar Medical University | 35(20) | 3014-3015 | Wang DM |
| 75 |  |  | Journal of Nanjing Medical University  (Natural Sciences) | 34(3) | 400-401 | Zhan F |
| 76 |  |  | Chinese Journal of Otorhinolaryngology Head and Neck Surgery | 49(1) | 69-70 | Zhao JG |
| 77 |  |  | Jilin Medical Journal | 35(5) | 1117-1119 | Yan K |
| 78 | 2015 | 3 | Practical Journal of Medicine & Pharmacy | 31(8) | 1366 | Hu JL |

Table 2.1 The articles of literature review in CNKI, VIP, and Wanfang database (continued 3)

|  | Year | No. | Journal | Volume (issue) | Page | Author |
| --- | --- | --- | --- | --- | --- | --- |
| 79 |  |  | Chinese Journal of Endocrine Surgery | 9(4) | 329-330 | Liu ZG |
| 80 |  |  | Compilation of Papers from the 2015 Zhejiang Ultrasound Medicine Academic Annual Conference | N/A | 152 | Zhang YY |
| 81 | 2016 | 3 | Journal of Clinical Otorhinolaryngology Head and Neck Surgery | 30(17) | 1406-1407 | Zhang RX |
| 82 |  |  | Cancer Research and Clinic | 28(12) | 840-842 | Wei YX |
| 83 |  |  | Clinical Misdiagnosis & Mistherapy | 29(2) | 37-38 | Liu YF |
| 84 | 2017 | 1 | Practical Oncology Journal | 31(2) | 156-158 | Zhang Q |
| 85 | 2018 | 3 | Journal of Shandong University(Health Sciences) | 56(7) | 90-91 | Qi RL |
| 86 |  |  | Chinese Journal of Ultrasound in Medicine | 34(6) | 575 | Yu L |
| 87 |  |  | Chinese Journal of Laboratory Diagnosis | 22(2) | 247 | Zhao SB |
| 88 | 2019 | 3 | Compilation of Papers from the 5th National Interventional Ultrasound Medical Academic Exchange Conference of the Chinese Society of Ultrasound Medical Engineering | N/A | 207 | Mu WN |
| 89 |  |  | Fudan Journal | 46(6) | 834-836 | Wang YY |
| 90 |  |  | Chinese Journal of Medical Imaging | 27(7) | 533-537 | Qu BQ |
| 91 | 2020 | 3 | National Medical Journal of China | 100(36) | 2867-2869 | Zhang XM |
| 92 |  |  | Chinese Journal of Operative Procedures of General Surgery | 14(6) | 647 | Xu L |
| 93 |  |  | Master's thesis of China Medical University | (1) | 1-23 | Feng ZJ |
| 94 | 2021 | 5 | Chinese Journal of Pathology | 50(11) | 1281 | Ye X |
| 95 |  |  | Chinese Journal of Pathology | 50(7) | 808-810 | Guan F |
| 96 |  |  | Chinese Journal of Current Advances in General Surgery | 24(9) | 755-756 | Zhou GL |
| 97 |  |  | Chinese Journal of Laboratory Diagnosis | 25(3) | 445-446 | Zhang GX |
| 98 |  |  | Journal of Clinical Otorhinolaryngology Head and Neck Surgery | 35(12) | 1132-1134 | Hu C |

N/A, not available.

Table 2.2 The articles of literature review in PubMed, Embase, Web of Science, and ProQuest database

|  | Year | No. | Journal | PMID/DOI/URL | Author | |
| --- | --- | --- | --- | --- | --- | --- |
| 1 | 1971 | 1 | Am J Clin Pathol. | 5099785 | Huang TY | |
| 2 | 1977 | 1 | J Surg Oncol. | 338999 | Kampsen EB | |
| 3 | 1980 | 1 | Cancer. | 7427886 | Shimaoka K | |
| 4 | 1981 | 1 | Cancer. | 7296515 | Saito K | |
| 5 | 1983 | 1 | Hum Pathol. | 6629365 | Motoyama T | |
| 6 | 1984 | 1 | Head Neck Surg. | 6469655 | Segal K | |
| 7 | 1985 | 1 | Jpn J Surg. | 3990051 | VK Kapoor | |
| 8 | 1988 | 2 | Jpn J Surg. | 3172590 | J Misonou | |
| 9 |  |  | Am J Surg. | 3394892 | Simpson WJ | |
| 10 | 1989 | 2 | Ann Otol Rhinol Laryngol. | 2910191 | GS Korovin | |
| 11 |  |  | Nihon Naika Gakkai Zasshi. | 2625600 | T Onai | |
| 12 | 1993 | 1 | J Laryngol Otol. | 7507159 | Theander C | |
| 13 | 1999 | 2 | Acta Cytol. | 10579006 | Mai KT | |
| 14 |  |  | Acta Cytol. | 10432891 | Kumar PV | |
| 15 | 2000 | 2 | The Ulster Medical Journal. | 10881647 | J M Jones | |
| 16 |  |  | Mod Pathol. | 10912933 | Kleer CG | |
| 17 | 2001 | 1 | Histopathology. | 11532039 | K Y Lam | |
| 18 | 2002 | 3 | Diagn Cytopathol. | 12357501 | Maheswar Sahoo | |
| 19 |  |  | Eur J Surg Oncol. | 11869012 | Zhou XH | |
| 20 |  |  | J Endocrinol Invest. | 12240907 | Kebapci N | |
| 21 | 2003 | 1 | Mil Med. | 12636140 | Zimmer PW | |
| 22 | 2004 | 1 | Endocrinologist | 10.1097/01.ten.0000114231.72375.3f | Mikhail Smirnov | |
| 23 | 2005 | 2 | J Clin Pathol. | 15917423 | Sutak J | |
| 24 |  |  | Endocrinologist | 10.1097/01.ten.0000175829.08167.26 | Chandna A | |
| 25 | 2006 | 4 | Surg Today. | 16440166 | Kitahara S | |
| 26 |  |  | Korean J Intern Med. | 16646570 | Tae Sik Jung | |
| 27 |  |  | Pediatr Dev Pathol. | 17163793 | Sergio Sanchez | |
| 28 |  |  | World J Surg Oncol. | 16984659 | Rohan R | |
| 29 | 2007 | 3 | Int Semin Surg Oncol. | 17397523 | Chintamani | |
| 30 |  |  | Surgeon. | 17849961 | Ab Hadi I | |
| 31 |  |  | Tumori. | 18038891 | Fassan M |  |
| 32 | 2008 | 4 | Pathol Int. | 19067856 | Tae Ik Eom |  |

Table 2.2 The articles of literature review in PubMed, Embase, Web of Science, and ProQuest database

(continued 1)

|  | Year | No. | Journal | PMID/DOI/URL | Author |
| --- | --- | --- | --- | --- | --- |
| 33 |  |  | Endocr J. | 18379125 | Makay O |
| 34 |  |  | Korean Journal of Otorhino-  laryngol Head and Neck Surg. | https://www.webofscience.com/wos/alldb/full-record/KJD:ART001291301 | Young H J |
| 35 |  |  | Intern Med J. | 18190421 | Müssig K |
| 36 | 2009 | 2 | Laryngoscope. | 19117291 | Long JL |
| 37 |  |  | Laryngoscope. | 10.1002/lary.20408 | Alexander |
| 38 | 2010 | 7 | Pathol Res Pract. | 19493635 | Rausch |
| 39 |  |  | Neth J Med. | 20508272 | Yucel H |
| 40 |  |  | Revista argentina de endocrinologíay metabolism. | https://www.webofscience.com/wos/alldb/full-record/SCIELO:S1851-30342010000400006 | Rodríguez |
| 41 |  |  | Tumori. | 20845818 | Luca R B |
| 42 |  |  | J Ultrasound Med. | 20966481 | Chia Y C |
| 43 |  |  | J. Afr. Cancer. | 10.1007/s12558-010-0100-6 | M Soufi |
| 44 |  |  | J Korean Surg Soc. | 10.4174/jkss.2010.78.1.55 | Kwak Jin |
| 45 | 2011 | 3 | Yeungnam Univ J Med. | https://www.webofscience.com/wos/alldb/full-record/KJD:ART002246075 | Ho Su K |
| 46 |  |  | ISRN Otolaryngology. | 23724255 | Meir W |
| 47 |  |  | J Bronchol Interv Pulmonol. | 23169089 | Batchelor |
| 48 | 2012 | 9 | Pathol Int. | 22192803 | Young S K |
| 49 |  |  | J Clin Oncol. | 22649151 | Chen KH |
| 50 |  |  | Head Neck. | 20652981 | De Vos |
| 51 |  |  | Korean Journal of Otorhino-  laryngol Head Neck Surg. | https://www.webofscience.com/wos/alldb/full-record/KJD:ART001629132 | Jeong S C |
| 52 |  |  | BMJ Case Rep. | 23220838 | William D |
| 53 |  |  | Head Neck Oncol. | 22452749 | Tunio MA |
| 54 |  |  | Auris Nasus Larynx. | 21855238 | Mercante |
| 55 |  |  | Cytopathology. | 21114558 | Rosa M |
| 56 |  |  | J Thyroid Res. | 21941683 | Yasuhiro I |

Table 2.2 The articles of literature review in PubMed, Embase, Web of Science, and ProQuest database

(continued 2)

|  | Year | No. | Journal | PMID/DOI/URL | Author |
| --- | --- | --- | --- | --- | --- |
| 57 | 2013 | 4 | Head Neck. | 23002023 | Shrestha M |
| 58 |  |  | Intern Med. | 23857092 | Ji In Lee |
| 59 |  |  | Indian J Palliat Care. | 24347911 | Ghoshal S |
| 60 |  |  | Korean Journal of Otorhinolaryngology Head and Neck Surgery. | https://www.webofscience.com/wos/alldb/full-record/KJD:ART001761191 | Seung H W |
| 61 | 2014 | 7 | Endocrinol Nutr. | 24332292 | Katty M |
| 62 |  |  | J Surg Case Rep. | 25487371 | Sapalidis K |
| 63 |  |  | Korean J Pathol. | 25588631 | Son Da H |
| 64 |  |  | Clin Nucl Med. | 24999700 | Cai Liang |
| 65 |  |  | J Otolaryngol Head Neck Surg. | 24942336 | Justin T L |
| 66 |  |  | Ultrasonography. | 24936508 | Ja Yoon J |
| 67 |  |  | Case Rep Pathol. | 25295208 | Bolfi F |
| 68 | 2015 | 2 | Head Neck. | 25491252 | Ryan N |
| 69 |  |  | Case Rep Pathol. | 26221558 | Chavan RN |
| 70 | 2016 | 4 | Auris Nasus Larynx. | 26589365 | Su Dong |
| 71 |  |  | Rom J Morphol Embryol. | 27833978 | Lichiardopol |
| 72 |  |  | Pathology. | 27318502 | Chu TP |
| 73 |  |  | Indian J Surg Oncol. | 27872538 | Vijendra S |
| 74 | 2017 | 5 | Endokrynol Pol. | 28879652 | Andrzej W |
| 75 |  |  | Rev Esp Med Nucl Imagen Mol. | 28219646 | L Caballero |
| 76 |  |  | BMJ Case Rep. | 28100571 | Michael DR |
| 77 |  |  | Int J Surg Case Rep. | 28633125 | Florian S |
| 78 |  |  | Mol Clin Oncol. | 28123737 | Tomoyasu Y |
| 79 | 2018 | 6 | Eur Ann Otorhinolaryngol Head Neck Dis. | 29914738 | Kallel S |
| 80 |  |  | Iranian Journal of Otorhinolaryngology. | 29387667 | Mohd I S |
| 81 |  |  | Auris Nasus Larynx. | 28739190 | Yasumatsu |
| 82 |  |  | Case Rep Oncol. | 30057536 | Satoshi K |
| 83 |  |  | Cytojournal. | 30294355 | Katie D |

Table 2.2 The articles of literature review in PubMed, Embase, Web of Science, and ProQuest database

(continued 3)

|  | Year | No. | Journal | PMID/DOI/URL | Author |
| --- | --- | --- | --- | --- | --- |
| 84 |  |  | Applied Radiology. | https://www.proquest.com/scholarly-journals/squamous-cell-carcinoma-thyroid-with-lymph-nodal/docview/2158433435/se-2?accountid=44440 | Ankita C |
| 85 | 2019 | 3 | Clin Ter. | 31304506 | De Cesare A |
| 86 |  |  | Ochsner J. | 31528145 | Raggio B |
| 87 |  |  | Gland Surg. | 32042676 | Wenlong W |
| 88 | 2020 | 8 | Scottish Medical Journal. | 32397900 | Alison E L |
| 89 |  |  | Onco Targets Ther. | 32021300 | Si Si Wang |
| 90 |  |  | Gland Surg. | 32420277 | Sun BH |
| 91 |  |  | Pathol Res Pract. | 32853962 | Mary T |
| 92 |  |  | Front Endocrinol (Lausanne). | 32849297 | Chen S |
| 93 |  |  | J Med Sci. | 10.4103/jmedsci.jmedsci_63_20 | Deshmukh |
| 94 |  |  | Int J Surg Case Rep. | 32416481 | Ramadhan T |
| 95 |  |  | Front Surg. | 33195392 | Zheng RZ |
| 96 | 2021 | 8 | BMJ Case Rep. | 34785519 | Sarah Akbar |
| 97 |  |  | BMJ Case Rep. | 33649031 | Michael M |
| 98 |  |  | Cureus. | 34123660 | Soror NN |
| 99 |  |  | Auris Nasus Larynx. | 34226099 | Sho Iwaki |
| 100 |  |  | BMJ Case Rep. | 33509876 | Joshua A |
| 101 |  |  | J Int Med Res. | 33827322 | Xin S |
| 102 |  |  | Eur Thyroid J. | 34956922 | Brandenburg |
| 103 |  |  | Eur Thyroid J. | 34950602 | Iwamoto Y |
